# Larval zebrafish as a model for studying individual variability in translational neuroscience research

**DOI:** 10.3389/fnbeh.2023.1143391

**Published:** 2023-06-23

**Authors:** Elina A. K. Jacobs, Soojin Ryu

**Affiliations:** ^1^Institute of Human Genetics, University Medical Center of Johannes Gutenberg University Mainz, Mainz, Germany; ^2^Living Systems Institute, Faculty of Health and Life Sciences, University of Exeter, Exeter, United Kingdom

**Keywords:** zebrafish, individual variability, translational neuroscience, neural circuit, *in vivo* imaging

## Abstract

The larval zebrafish is a popular model for translational research into neurological and psychiatric disorders due to its conserved vertebrate brain structures, ease of genetic and experimental manipulation and small size and scalability to large numbers. The possibility of obtaining *in vivo* whole-brain cellular resolution neural data is contributing important advances into our understanding of neural circuit function and their relation to behavior. Here we argue that the larval zebrafish is ideally poised to push our understanding of how neural circuit function relates to behavior to the next level by including considerations of individual differences. Understanding variability across individuals is particularly relevant for tackling the variable presentations that neuropsychiatric conditions frequently show, and it is equally elemental if we are to achieve personalized medicine in the future. We provide a blueprint for investigating variability by covering examples from humans and other model organisms as well as existing examples from larval zebrafish. We highlight recent studies where variability may be hiding in plain sight and suggest how future studies can take advantage of existing paradigms for further exploring individual variability. We conclude with an outlook on how the field can harness the unique strengths of the zebrafish model to advance this important impending translational question.

## Introduction

For decades, the reductionist approach has been one of the most successful in biological research: by reducing as much as possible any confounding factors arising from environmental differences, and even genetic background, we have managed to isolate the effects of a factor of interest. While this approach certainly has its merits and has produced many important discoveries, variability is part of nature. In 1973, geneticist Dobzhansky coined the famous phrase “Nothing in biology makes sense except in the light of evolution.” The traditional reductionist approach seeks to minimize a critical aspect of evolutionary relevance: variability. Without variability, natural selection has no substrate to act on, and without variability, a species has less chance of survival in case of environmental changes (Xue et al., 2019).

An important source of variability is genetic variation across a population, which comprises different distributions and combinations of different alleles of given genes across individuals. Most research investigating genetic factors in variability focus on the genetic effects on trait means: assuming that if two alleles for example have different effects on a trait, this results in different trait means. However, recent evidence suggests that phenotypic variability itself may be under genetic control: two alleles may have the same trait means but differing variances, meaning one allele may be associated with more phenotypic extremes than another (Bruijning et al., 2020; Maloney, 2021). In a typical outbred population such as the human population, where each individual is a unique instance of a particular genotype, investigating and estimating the effects of intragenic variability would be impossible. But in inbred experimental models such as *Drosophila*, distinguishing variance that stems from a single gene from variability across genes, and how this contributes to variation seen amongst individuals, becomes tractable. Such research has revealed that several behaviors, including turn bias during navigation in a Y-maze, overall activity, and turn regularity, show significant genetic control of variation (Ayroles et al., 2015). Importantly, the variances across traits were uncorrelated, suggesting independent loci controlling variance within these traits. These results are remarkable if pertinent to humans, as even the most well-powered GWAS studies have failed to produce unequivocal clues about the causative neurobiological processes implicated in psychiatric disorders (Horwitz et al., 2019). An association study of handedness in *Drosophila* found many genes with expression in adult and larval CNS, including a synaptic target recognition gene that is highly conserved (Ayroles et al., 2015), and intragenic variance has also been observed in several other species (Bruijning et al., 2020). These observations provide a promising indication that the results may have broad applicability. If trait variance is under genetic control in humans, then individuals with the same genotype may be perfectly healthy if their trait expression is close to the trait mean, or show completely different forms of pathologies depending on which extreme of the distribution they fall onto. This may also explain why individuals with known genetic markers can have entirely different pathologies (Chubb et al., 2008).

Despite decades of research, no consistent indications for disease etiology have emerged for depression or other psychiatric diseases (Treasure et al., 2015; Pandarakalam, 2018; Vieta et al., 2018; Potenza et al., 2019; Moncrieff et al., 2022; Stein et al., 2022). However, if intragenic variance plays a role in human neuropsychiatric diseases, more detailed investigations into not just trait means but also trait variances will be indispensable for furthering our understanding of disease etiology and effective treatments for different individuals. A useful starting point for this endeavor lies in the comparison of human individual differences studies with studies from model systems. If individual differences in a given behavior are observable in humans and similar differences occur in a model organism in which these can be attributed to intragenic variance, this may provide clues to disease etiology when a candidate gene approach reveals no differences in trait means. We suggest that a part of the answer to current difficulties in neuropsychiatric research may lie in this approach and argue that we should pay closer attention to variability and individual differences in translational neuroscience. In this mini-review after giving case examples of studying individual variability, we discuss the potential of larval zebrafish for studying individual variability in context of neural circuit analysis and translational neuroscience.

## Examples of the importance of individual differences

Human neurobiology textbooks famously illustrate the lateralization of language processing in the left cortical hemisphere using the seminal discoveries of Broca and Wernicke. Yet, recent research has shown that the classic narrative of language as a prime example of lateralization and specialization is not as simple (Johns et al., 2008; Friederici, 2011; Chang et al., 2015). Asymmetry in language representation across left and right hemisphere correlates with reading skill: while highly skilled readers show the typical left hemisphere activation during language processing, less skilled readers show activation in the right hemisphere (Prat et al., 2007; Prat et al., 2011; Chiarello et al., 2012). These differences have important clinical relevance: not all stroke patients with the same stroke localization will display the same impairments (Knecht et al., 2002). Language is arguably a uniquely human feature and difficult if not impossible to study in animal models and gain translational insights from. Nevertheless, language lateralization correlates with several other cognitive and behavioral features that can more easily be assessed in other species. Specifically, language lateralization correlates with the degree of motor cortex lateralization, which in turn is strongly influenced by the degree of hand-preference (Knecht et al., 2000). This is of special interest, as one of the behaviors investigated in the study of intragenic variance was a measure of “handedness” as defined by the preference for turning left or right in a Y-maze (Ayroles et al., 2015). Interestingly, in humans handedness is a trait that shows considerable variability: although it is often considered binary, with individuals either categorized as right or left handed, handedness lies on a continuum, with up to 35% of the population being ambidextrous to varying degrees (Knecht et al., 2000). Additionally, handedness correlates with visual processing, with differences in lateralization of facial processing across individuals (Willems et al., 2010; Frassle et al., 2016). Altogether, these examples show how neural representations depend on individual characteristics in common every-day processes but that are typically studied in a “single solution” fashion.

Yet, this variability frequently gets overlooked. For example, human neuroimaging studies frequently focus on right-handers, either excluding left-handed or ambidextrous participants, or grouping ambidextrous individuals with a right-hand preference together with strongly right-handed individuals. This is unfortunate, as this likely misses or masks differences in the degree of lateralization of brain functions, which could equally have important clinical implications in cases of brain trauma or disease. Indeed, in some cases, averaging across individuals without assessing individual variability may lead to a null-result, where no neural representations are uncovered, when in fact they may exist but depend on non-overlapping individual differences (Alfred et al., 2021).

A great example from animal research demonstrating the importance of understanding individual differences and the role that neuromodulators play in them comes from work in the crustacean stomatogastric ganglion (Hamood and Marder, 2014; Daur et al., 2016; Marder et al., 2022). This constitutes a comparatively small circuit of 30 neurons that are well-characterized and identifiable across animals, and produce known outputs like the pyloric rhythm, which drives the digestion in these animals. One might expect that a circuit like this would be rigid in its properties compared to the brain, but even this comparatively “simple” circuit has a lot of room for variability, and multiple solutions for creating stereotyped circuit outputs. A combination of experimental and theoretical work has revealed that similar behavior at the level of single neurons can result from very different combinations of electrophysiological properties, and that similar network performance can in turn be produced by varying combinations of synaptic and cellular properties within the network (Prinz et al., 2004; Goaillard et al., 2009). Importantly, the effects of neuromodulators on circuit activity depend on the combination of cellular parameters that are present in a given circuit, and can thus vary from small to dramatic in different circuits with otherwise similar behaviors (Goldman et al., 2001). This observation provides possible clues about the varying response and tolerability of pharmacological agents between individuals, including individuals being treated for neuropsychiatric disorders. Additionally, differences in cellular properties were shown to correlate with differences in molecular characteristics (Goaillard et al., 2009). Together, these results suggest that seeking to understand the molecular properties alone of neurons within a circuit has limited predictive power of circuit behavior, as the same neuronal properties will lead to different outcomes depending on the properties of other neurons in the circuit, and conversely, that neurons with very different molecular characteristics can produce similar, functional circuit outputs. These insights are particularly relevant as the animals that generated these results are not laboratory-raised, but wild-caught animals that were brought to the lab. This means that each individual, with its own variations, has succeeded in its environment until the point it was caught (Nassim, 2018). Taken together, these results provide a powerful example of how there can be considerable variability at the level of neural circuits that contributes to the success of a species and constitutes viable solutions.

## Studying individual differences in larval zebrafish

The larval zebrafish is a powerful model for investigating the neural basis of behavior due to the possibility of *in vivo* whole-brain imaging at cellular resolution at the same time as larvae are engaged in a behavior. Many studies have used this system to make compelling advances in our understanding of how neural circuits produce behavioral output. However, given the variability within even relatively simple behaviors, it is possible that biologically viable differences in solutions for producing adequate behaviors are missed by not investigating variability more explicitly. Zebrafish lines are typically not isogenic, which may limit how much researchers can learn about the sources of variability, although the generation of isogenic lines is possible in principle, albeit technically demanding (Franěk et al., 2019; Adams et al., 2020). Nevertheless, maintaining genetic diversity can be advantageous depending on the study, as this more closely resembles human populations. The investigation of variability is further complicated by the fact that studies often employ different strategies for dealing with variability.

### Common strategies for dealing with variability in current zebrafish neural circuits research

**FIGURE 1:**
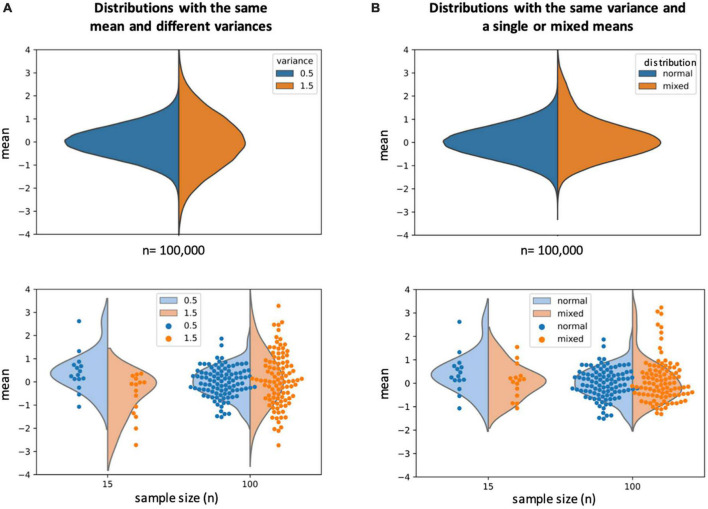
**(A)** Top: illustration of two “ideal” normal distributions with the same mean (0) and different variances (blue = 0.5, orange = 1.5). Bottom: two examples of different sample sizes drawn from the distributions illustrated at the top. A small (*n* = 15) sample size may fail to reveal the differences in variances and even erroneously indicate differences in the mean, when in actuality both samples are drawn from distributions with the same mean but different variances. A larger sample size (*n* = 100) more accurately represents both the mean and variance of the population the samples were drawn from. **(B)** Top: illustration of a normal distribution (blue) and a distribution that results from a combination of two distributions with different means (orange). When only a small portion of the sample (here 10%) comes from a distribution with a different mean, this is difficult to detect in the average. Bottom: a small sample size may not reveal any samples that deviate from the majority, while a larger sample size can make outliers more obvious and detectable. 10% of a sample can appear like a small component that may get discarded as outliers, but it is estimated that 5–10% of the human population is left-handed (Knecht et al., 2000). This percentage is high enough that most people know at least one left-handed person, and discarding these outliers would be akin to neglecting the existence of left- handed individuals.

A common first step in analysis pipelines is the alignment of neural data from individual fish to a common framework. This makes individual samples more easily comparable to each other, and renders the results more comparable to previously published results as well. But after this step, methodologies frequently diverge. Some studies combine the data from all fish into one large dataset and only exclude individuals for technical reasons such as poor data quality. See for example (Heap et al., 2018; Andalman et al., 2019; Bahl and Engert, 2020; Oldfield et al., 2020; Marquez-Legorreta et al., 2022). This strategy maximizes statistical power, but forfeits information about individual fish and any possible differences and the results are usually presented as averages. This method risks generating an average result that is not present in any individual, although this can easily be accounted for by verifying if a representative result is present at the individual level, which many studies indeed do. The more likely risk is that a less common result is averaged out and not detected, even though it may represent a true biological solution (see Figure 1 for an illustration). Heap et al. (2018) and Andalman et al. (2019) performed elegant studies investigating the neural correlates of looming dot and inescapable adverse stimulus responses, respectively. Both studies relied on the method of combining data across all individuals. Yet, these behaviors share similarities with the acoustic startle behavior investigated by Pantoja et al. (2016, 2020), which showed stable individual differences that correlated with differences in neural activities. It would therefore be valuable to explicitly investigate the possibility of individual differences in these behaviors and possible differences in neural strategies in future studies.

Other studies only include data from fish if the neural responses are replicated in at least *X* fish, with the threshold *X* varying from a seemingly arbitrary minimum number to the maximum possible number of fish. See for example (Kawashima et al., 2016; Chen et al., 2018; Haesemeyer et al., 2018; Marques et al., 2020; Vanwalleghem et al., 2020). Chen et al. (2018) and Marques et al. (2020) used this method for revealing convergent neural sensorimotor representations and the neural correlates of state switches between exploration and exploitations, respectively. This method successfully identifies the most commonly employed biological strategy, but risks interpreting this as a universal solution when there may in fact be more nuance or alternative solutions that were discarded in the sample selection process. Marques et al. (2020) for example mention that a subset of their fish were better described by a 3 rather than 2 state model, an observation that represents a promising avenue for further investigating how such differences across individuals may arise.

Lastly, some studies not only apply a selection criteria to neural responses, but also only investigate the neural responses of larvae that meet certain selection criteria in their behavior (Kawashima et al., 2016; Mu et al., 2019; Bahl and Engert, 2020; Lin et al., 2020). This method facilitates high specificity about which neural circuits are involved in a given behavior. Yet, information about individual differences and to what extend behavioral differences correlate with neural differences is lost. For example, Mu et al. (2019) performed a beautiful study investigating the neural mechanisms of passive coping in response to futile attempts of swimming against a fictive current. However, only half of the fish exhibited the investigated passive state, the rest continued to try to swim. A future study comparing the neural activity between fish that do and do not exhibit the passive state could establish to what extend the neural states found in the passive fish are a universal signature of passivity, in particular if optogenetically inducing such states in the brains of fish that previously did not become passive then activates passivity in these fish. Lin et al. (2020) investigated heat aversion and taught larval zebrafish to turn in a specific direction to turn off heat. Only 39% of larvae learned the full task, which included blocks in which the turn directions were switched, 15% of larvae learned to turn in one direction but failed to learn the reversal, and 46% did not learn the task at all. The analysis of the neural responses from the learner fish revealed several clusters of neurons related to the task, with most clusters coinciding with anatomical structures, but also many neurons outside the main anatomical clusters, suggesting widespread representations. Future studies comparing the neural activity between the different types of learners could reveal more detailed information about the role of the different anatomical clusters and whether they are universally related to learning, or whether different brains have different strategies.

All of these studies are prime examples of the reductionist method, which seeks to minimize variability to reveal an underlying general principle. Undoubtedly this method has produced invaluable insights into neural function. However, given the rapid progress in methods and increase in studies investigating *in vivo* behavior and neural physiology, we are now at a point where investigating individual differences and the inherent flexibility in neural networks that this reveals becomes feasible. For a recent review on the topic of neural variability and its relevance for behavior, readers can refer to Waschke et al. (2021).

### Examples of studies of individual differences using larval zebrafish

Although several individual differences studies exist in adult zebrafish, we will focus here on studies in larval zebrafish that combine investigations at the behavioral and neural level. Of particular interest is recent work from Horstick et al. (2020), which demonstrated that zebrafish larvae show similar asymmetrical motor preferences to what was observed in the intragenic variance study in *Drosophila* (Ayroles et al., 2015). In the absence of visual stimulation, zebrafish larvae exhibited left or right “handedness” by preferentially turning in one or the other direction during dark-induced circling. The preference was consistent across trials for individual larvae, while across the population there was no average tendency toward one or the other direction. Importantly, inbreeding of individuals with the same motor bias did not result in progeny with the same bias but a distribution that was balanced on average, suggesting that the bias is not heritable. Interestingly, follow-up work showed that this “handedness” lies on a continuum, with more or less biased individuals (Hageter et al., 2021), similar to what is seen in humans. The authors identified neurons in the posterior tuberculum that project to habenula as drivers of the motor asymmetry, and further showed the development of this asymmetry is sensitive to temperature but not to other environmental factors such as exposure to conspecifics, enrichment, salinity or physical disturbance. Lastly, Notch signaling, a highly conserved pathway involved in neurogenesis (Sprinzak and Blacklow, 2021), is required for the development of this motor asymmetry. This work provides a promising avenue for investigating individual differences in zebrafish, a model in which neural representations can be studied *in vivo* at cellular resolution in the whole brain, and where it is possible to control genetic backgrounds and investigate different sources and mechanisms of variability.

In an example involving sensory processing, Pantoja et al. (2016) showed that larval zebrafish show stable individual differences in habituation rates to acoustic startle stimuli. The acoustic startle response (ASR) is an innate defensive response to sudden noise, and here the authors investigated the role of neuromodulatory regulation, as the neuronal populations involved in the ASR receive extensive neuromodulatory inputs. This is of particular interest: both alterations in this type of sensory processing as well as neuromodulatory circuits are associated with neuropsychiatric disorders (Braff et al., 1992; Lopez-Schier, 2019). Pantoja et al. (2016) found that activity in the serotonergic dorsal raphe nucleus negatively correlated with escape probability in response to the acoustic startle stimulus: the more serotonergic neurons were active in a given trial, the lower the probability of escape, and with habituation, the number of active neurons decreased. In animals that habituated faster, the activity in these neurons decreased faster than in animals that habituated more slowly. Investigations into the role of dopamine revealed an opposite effect to serotonin: the more dopaminergic input, the faster the habituation. In contrast to the turning behavior mentioned above, creating F1 progeny from individuals habituating quickly or slowly resulted in populations that overall habituated more quickly or slowly, respectively, suggesting that this behavioral pattern is heritable. Nevertheless, considerable variation amongst the two types of F1 progeny remained, and importantly, serotonergic activity did not perfectly predict escape probability and habituation rates. In some individuals high serotonergic activity was also accompanied by high rates of habituation, suggesting that whilst serotonergic modulation is involved in the process, there are other contributing factors, and the overall pattern of behavior likely depends on a combination of factors. This point is further illustrated in a follow-up study that looked at the patterns of brain activity from the progeny of either low or high habituating individuals, and found that there are differences in multiple brain regions that correlate with these behavioral differences (Pantoja et al., 2020). These studies provide powerful examples of how looking at individual differences can provide further understanding of circuit mechanisms contributing to different behaviors.

### Potential approaches for studying variability using larval zebrafish

Currently, sample sizes in larval zebrafish imaging studies are small; n is usually reported anywhere between 6 and 30. Investigating individual differences, and establishing whether any differences in neural solutions represent experimental artifacts or true biological variability, will require collecting much larger sample sizes; estimates from human neuroimaging studies of individual differences suggest sample sizes of *n* > 100 (Dubois and Adolphs, 2016). The zebrafish model is often lauded for its scalability to larger sample sizes, given the large numbers of larvae that can be obtained and their small size, which makes their maintenance easy and cost-effective. However, the simultaneous recording of neural activity and behavior represents a bottleneck, as often larvae need to be acclimatized to the imaging set-up to observe a behavior in a gel-fixed set-up, and the slow calcium dynamics require imaging over longer periods of time with many repetitions. Nevertheless, given the rapid progress in imaging technologies, particularly in the domain of light-sheet and light-field imaging (Hillman et al., 2019; Zhang et al., 2021), which provide ever larger sampling rates of whole-brain imaging, as well as the continued refinement of fluorescent reporters of neural activity (Lin and Schnitzer, 2016; Zarowny et al., 2020), which can easily be implemented in transgenic zebrafish, we argue that the larval zebrafish is still the most suitable model to pursue this challenge. First, it is feasible that the ease and speed of data collection will continue to increase, in which case approaching sample sizes of 100 + in larval neuroimaging studies will not be prohibitively expensive and/or time-consuming. Second, even if achieving large sample sizes may remain challenging for small research groups, a possible solution is to collaborate across laboratories and utilize multi-lab data repositories. This approach is commonly used in human neuroimaging and genetics research to achieve large sample sizes, and is also implemented in rodent research, for example The International Brain Laboratory (2017). Another possibility is to take advantage of the scalability of experiments at the behavioral level and to use the behavioral data to select different extremes in behavior for comparison in smaller numbers at the neural level (De Haas, 2018). The larval zebrafish model is particularly well suited for this approach given the possibility of multi-well imaging of up to 96 larvae simultaneously, for which automated behavioral analysis techniques are available (Creton, 2009). Indeed, this is the approach taken by Pantoja et al. (2020), and it is not dissimilar from approaches comparing standard and disease-model samples, as was done by Marquez-Legorreta et al. (2022). This approach is also particularly suitable for high-throughput pharmacological screens for drug discovery (Baxendale et al., 2017). Although this approach has its pitfalls – variability across a sample may not be linear, and extremes may be associated with starker differences than less extreme variations – it will be a step toward creating a fuller understanding of how neural circuits generate different types of behavior.

Lastly, once larger sample sizes are achieved, novel analysis tools such as factor analytic, clustering, and modeling approaches such as the ANN analysis by Andalman et al. (2019) will be instrumental in understanding the prevalence and functional role of any differences in neural responses across different individuals.

## Conclusion

The larval zebrafish model provides a unique opportunity for gaining insights in translational neuroscience, particularly in the neural circuits domain due to the possibility of recording whole-brain cellular resolution *in vivo* data. The combination of systems and reductionist approaches has provided compelling insights into neural function. However, the reductionist approach often implicitly assumes that there is a single, perhaps ‘optimal’ solution, when in reality it is likely that evolution has produced several ‘good enough’ solutions (Holmes and Patrick, 2018). Research from human neuroimaging and other model organisms has begun to reveal the translational relevance of individual variability, and we have provided examples of how we can harness the strengths of the larval zebrafish model to contribute to this important emerging field. Personalized medicine is an ambitious goal, but with the right approach, zebrafish can provide insights to get closer to it.

## Author contributions

EJ conceptualized and wrote the first draft of the manuscript. SR edited the manuscript. Both authors revised, read, and approved the manuscript.
